# High-throughput phenotyping platform for analyzing drought tolerance in rice

**DOI:** 10.1007/s00425-020-03436-9

**Published:** 2020-08-10

**Authors:** Song Lim Kim, Nyunhee Kim, Hongseok Lee, Eungyeong Lee, Kyeong-Seong Cheon, Minsu Kim, JeongHo Baek, Inchan Choi, Hyeonso Ji, In Sun Yoon, Ki-Hong Jung, Taek-Ryoun Kwon, Kyung-Hwan Kim

**Affiliations:** 1 The National Institute of Agricultural Sciences, 370 Nongsaengmyeong-ro, Wansan-gu, Jeonju-si, Jeollabuk-do Republic of Korea; 2 Department of Agricultural Machinery Engineering, Chungnam National University, Daejeon, 34134 Republic of Korea; 3 Department of Crop Science and Biotechnology, Jeonbuk National University, Jeonju, 54896 Republic of Korea; 4 Graduate School of Biotechnology and Crop Biotech Institute, Kyung Hee University, Yongin, Republic of Korea

**Keywords:** Drought stress, RGB, NIR, IR, Fluorescence, Parameter

## Abstract

**Main conclusion:**

A new imaging platform was constructed to analyze drought-tolerant traits of rice. Rice was used to quantify drought phenotypes through image-based parameters and analyzing tools.

**Abstract:**

Climate change has increased the frequency and severity of drought, which limits crop production worldwide. Developing new cultivars with increased drought tolerance and short breeding cycles is critical. However, achieving this goal requires phenotyping a large number of breeding populations in a short time and in an accurate manner. Novel cutting-edge technologies such as those based on remote sensors are being applied to solve this problem. In this study, new technologies were applied to obtain and analyze imaging data and establish efficient screening platforms for drought tolerance in rice using the drought-tolerant mutant *osphyb*. Red–Green–Blue images were used to predict plant area, color, and compactness. Near-infrared imaging was used to determine the water content of rice, infrared was used to assess plant temperature, and fluorescence was used to examine photosynthesis efficiency. DroughtSpotter technology was used to determine water use efficiency, plant water loss rate, and transpiration rate. The results indicate that these methods can detect the difference between tolerant and susceptible plants, suggesting their value as high-throughput phenotyping methods for short breeding cycles as well as for functional genetic studies of tolerance to drought stress.

**Electronic supplementary material:**

The online version of this article (10.1007/s00425-020-03436-9) contains supplementary material, which is available to authorized users.

## Introduction

The worldwide population is projected to increase to 90 billion people by 2050, and the availability of water is an important problem (Rockström et al. 2009; Ray et al. 2013). Global water shortages are becoming a topic of interest because they limit the production of most crops including rice. When the soil water content is below the saturation level during the growth of rice, production decreases by > 50% (Lafitte et al. 2007; Lesk et al. 2016). These issues have led to the development of drought-resistant cultivars, and techniques are being developed to accurately select them in a short period of time.

Plant phenomics technologies are being developed to characterize the morphological and physiological responses of plants using digital images (Furbank and Tester 2011; Großkinsky et al. 2015; Kumar et al. 2015; Perez-Sanz et al. 2017; Tardieu et al. 2017). Phenomics technology allows the study of seed size, leaf color, biomass, and fruit ripeness throughout plant development (Houle et al. 2010; Fiorani and Schurr 2013; Hurtado et al. 2013; Maloof et al. 2013; Gustin and Settles 2015; Vanhaeren et al. 2015). The response to abiotic stresses including drought stress has been investigated extensively in various crops (Harris et al. 2010; Saito et al. 2010; Gupta et al. 2012; Rebolledo et al. 2012; Hairmansis et al. 2014; Anupama et al. 2018; Spindel et al. 2018). For example, a phenotypic analysis of sorghum was performed by photographing shoots deprived of nitrogen and water. Data extracted from near-infrared (NIR) images include senescence, mean Hue angle, compactness, convex hull area, and eccentricity (Neilson et al. 2015). In spring barley exposed to drought stress, phenotypic traits were analyzed in detail, including geometric traits, color-related traits, fluorescence-related traits, and NIR-related traits (Chen et al. 2014). A genome-wide association study in rice analyzed 533 accessions by high-throughput phenotyping using red–green–blue (RGB) images (Yang et al. 2015). Image-based traits were extracted from 507 rice accessions were classified into biomass, greenness, morphological, and histogram texture (Guo et al. 2018). Duan et al. (2018) analyzed four RGB traits including green projected area ratio, total projected area area/bounding rectangle of area ratio, perimeter/projected area ratio, and total projected plant area/convex hull area ratio from 40 drought-resistant and drought-sensitive accessions of rice. Drought stress analysis platforms in rice are mostly based on RGB traits, and various imaging analysis techniques including RGB are needed to develop them.

Plant phenomics platforms are currently operated by governments and private companies in more than 14 countries (Kim et al. 2017). A recently built our platform included RGB, NIR, fluorescence imaging sensors, and an infrared (IR) camera, as well as software pipelines to quantify morphological and physiological measurements. High-throughput phenotyping system is a plant-to-sensor type and is designed to take images from the side and top-view of 1012 plants, and the XYZ system is a sensor-to-plant type and it can measure RGB, fluorescence, and 3D images from up to maximum 1800 plants in a sensor-to-plant type. In this study, to construct a phenotyping platform for drought in rice, eight image-based parameters including projected plant area, color, object extent X, object extent Y, convex hull area, compactness, eccentricity, and center of mass Y were extracted from RGB images. After image capture, the water content of the plant was analyzed based on NIR intensity (Eitel et al. 2006; Seelig et al. 2008), and NIR measurements were used to indicate plant biomass (Gitelson et al. 2003; Briglia et al. 2019). IR thermography was used as a phenotyping tool through temperature measurements of plants under drought stress conditions. Plants under water-deficit stress show a higher temperature in the leaf blade and sheath (Jones et al. 2009; James and Sirault 2012; Prashar and Jones 2014). Chlorophyll fluorescence (ChlF) is analyzed by quantitative evaluation of the response to abiotic stresses such as salt, heat, and drought (Barbagallo et al. 2003; Dobrowski et al. 2005; Woo et al. 2008; Mehta et al. 2010). We used the ChlF parameters F0, Fm, and Fv from the photochemical reaction of photosystem II (PSII) (Parkhill et al. 2001; Sharma et al. 2015). The Fv/Fm ratio was used as a representative abiotic stress physiological index (Guidi and DeglInnocenti 2011; Murchie and Lawson 2013; Kalaji et al. 2016). In addition, the water use efficiency (WUE) and transpiration rate (TR) were measured using the DroughtSpotter system to determine water availability and transpiration in plants according to drying time. In *Arabidopsis*, phytochrome B increases drought resistance by increasing abscisic acid sensitivity (Boccalandro et al. 2009; González et al. 2012). The rice phytochrome B (*OsPhyB*) gene is a flowering repressor in short-days and long-days, and it is a photoreceptor that recognizes red and far-red light (Ishikawa et al. 2009, 2011; Takano et al. 2009; Wang et al. 2010; Piao et al. 2015; Legris et al. 2016). *OsPhyB* is also involved in a drought-tolerant pathway in the roots in response to water deficiency (Yoo et al. 2017). In this study, the *osphyb* was used to digitally quantify drought traits, as well as physiological changes in response to drought in rice.

Here, we present a powerful platform for the early and quantitative determination of drought-tolerant phenotypes in rice. The use of various imaging techniques and parameters to accurately analyze drought traits aims to provide comprehensive information to improve our understanding of the responses of plants to drought. In addition, our platform can be developed into a high-throughput phenotyping system for analyzing digitized phenotypes.

## Materials and methods

### Plant materials and drought stress conditions

The wild type (WT) and *osphyb* seeds were used for experiments were obtained from Kyunghee university. The *osphyb* mutant line was a T-DNA knock-out line (4A-02226) in which T-DNA was inserted into the third intron of rice *phytochromeB* (LOC_Os03g19590). Seeds were sterilized and grown for 3 weeks after sowing in low salinity (< 0.05%) soil. Pots measuring 12 cm in diameter were filled with 330 g soil each and soaked in a water-filled bed for 24 h to maintain constant water content. Rice was grown under a 14 h light/10 h dark photoperiod in a greenhouse with controlled humidity at 50%; light intensity was approximately 1000 µmol/m^2^/s.

WT and *osphyb* seeds were divided into two groups; one group was grown under normal conditions, and the other was exposed to drought stress. 20 WT plants and 20 *osphyb* plants were used in the normal group, and 20 WT plants and 20 *osphyb* plants were also used in the drought group. WT and mutants were arranged in a cross array pattern to minimize differences in drought phenotype according to location on the conveyor. In the drought stress condition, the water content was measured daily using a soil moisture sensor (WP700 logger; Mirae Sensor, Seoul, Republic of Korea). Plants grown under normal conditions for 3 weeks showed a phenotype of drought stress, such as leaf wilting, first of all, with drought treatment reducing the water content in the soil to near 0%. At this time, the drought treatment was stopped and water was supplied again. When water was re-supplied, the plants sensitive to drying were difficult to recover, and the plants resistant to drought recovered and continued normal growth. In the drought stress group, the water content of the soil was measured at 11:00 am daily. The drought stress conditions mean that water supply to the soil is stopped after normal growth, and the soil water content is a percentage of the relative water content in the soil.

### RGB imaging and extraction of image-based parameters

WT and *osphyb* plants were photographed using a 3D scanalyzer imaging system with a 6576 × 4384 resolution RGB camera (LemnaTec, GmbH, Aachen, Germany) using constant light conditions, plant location, and camera settings (gamma, 65; gain, 1000; exposure time, 38,000 µs). Images were acquired from a side-view at 0°, 120°, and 240°. After obtaining RGB images, NIR images were taken in another imaging chamber. The captured images were converted into PNG files and analyzed using LemnaGrid software (LemnaTec GmbH, Aachen, Germany). The image-based parameters analyzed were as follows: projected plant area, plant color, convex hull area, compactness, eccentricity, object extent X and Y, and center of mass Y.

Plant color was analyzed using Hue channels from 0 to 180 regions to distinguish between plants grown under normal conditions and those grown under drought stress. Hue channels from regions 0–72 (yellow area) represented plants grown under drought stress, whereas those from regions 73–180 (green area) represented plants grown under normal conditions. Plant growth rate was calculated using the projected plant area as an RGB parameter. This value was calculated by dividing the projected plant area by time (day) in the two time periods used to calculate growth rates.

### Application of NIR imaging for measuring plant water content

The water content of plants was measured using a NIR camera (Model: Goldeye G032, AVT Allied Vision, Exton, USA) with a resolution of 636 × 508 pixels. NIR images were acquired at 0°, 120°, and 240° from a side-view. Because it is difficult to delineate the region of interest using NIR images, response to stress data were obtained by matching RGB and NIR images. To compensate for differences in the position of plants and image resolution between the two cameras, image matching was performed using the local matching method in 130 matching points. The matched images were cropped to the same size as the NIR images, and only the plant area calculated from the RGB image was extracted to confirm the average water content. The NIR intensities were obtained at the water absorption wavelength of 1450 nm; therefore, plants with a high water content showed a low NIR intensity. For NIR intensity, the final results were calculated as a reciprocal number for easy assessment of drought-related phenotypes.

### Application of thermal imaging for measuring plant temperature

IR (infrared) images were acquired from 3-week-old plants using the uncooled microbolometer focal plane array with a FLIR P620 camera (FLIR Systems Inc., North Billerica, MA, USA) from the beginning of the drought stress phase (DSP) to the re-watering phase (RWP). The IR resolution was 640 × 480 pixels, and the spectral range was 7.5–13 µm. The imaging camera was set up at a distance of 1.5 m from the plant, and the WT and *osphyb* plants were placed on the image capturing frame. This frame was covered with a 5 cm-thick styrofoam sheet to minimize changes in the background temperature, and the inside was covered with a black cloth. The filming room was maintained at a temperature of 26 °C and humidity of 30% during image acquisition. Thermal images were analyzed using FLIR Research IR 4.1 software. For thermal imaging, plants were exposed to drought stress by stopping water in the growth room, and water was re-supplied when the leaf wilting became severe. The photographs were taken at approximately 11:00 am, which is the period of high photosynthesis efficiency.

### Application of fluorescence imaging for measuring photosynthesis efficiency

The PlantScreen™ Robotic XYZ System (Photons Systems Instruments, Brno, Czech Republic) was used for fluorescence imaging to measure photosynthetic efficiency including maximum quantum yield (QY_max) during the DSP and RWP. Plants grown for 3 weeks under long-day conditions (14 h light/10 h dark) were transferred to a precision environment control room, and the water supply to the pots was stopped to induce drought stress. Water content measurements and the recovery process were performed as described for RGB imaging. The environmental control room was maintained under a 14 h light/10 h dark photoperiod with a daytime temperature of 30 °C and a night-time temperature of 25 °C. The humidity was set at approximately 20%, and the CO_2_ concentration was 700 ppm. The light source was LED with a mixture of white and red light (50% each), and the intensity was approximately 1000 µmol/m^2^/s. The fluorescence imaging system associated with the RGB camera had a resolution of 1392 × 1040 pixels. Plants were subjected to dark adaptation for 15 min before fluorescence imaging. F0, Fm, and Fv values were obtained to determine the photosynthetic efficiency according to chlorophyll fluorescence. RGB imaging was also performed from a top-view, and RGB image analysis was performed using ImageJ (https://imagej.net/Downloads) to analyze the plant area and perimeter during the DSP and RWP.

### Measurement of WUE, plant water loss rate, and transpiration rate

DroughtSpotter (Phenospex, Herleen, the Netherlands) was used to measure WUE, plant water loss rate (PWLR), and the TR of WT and *osphyb* plants under drought stress conditions. The precision environment room was placed under a 14 h light/10 h dark photoperiod, which is suitable for growing rice, and was maintained at a daytime temperature of 30 °C and a night-time temperature of 25 °C with 50% humidity. Plants were grown under these conditions for 3 weeks after sowing. The ‘deviation mode’ represented the conditions in which water was added when the water in the pots was inadequate for 7 days, and the weight limit of pots during irrigation was 800 g. Irrigation was carried out when 5% was insufficient at 800 g. The formula for calculating WUE was as follows (Karaba et al. 2007):

$$\begin{aligned} {\text{WUE}} = & \left[ {{\text{Dry weight after drought (g)}} - {\text{Dry weight before drought (g)}}} \right] \\ & /{\text{Total irrigated water (g)}} \\ \end{aligned}$$

The PWLR and TR were measured in the ‘none mode’ for 7 days after the weight of the pots reached 800 g without further water supply. The PWLR was calculated by subtracting the soil water loss rate from the total water loss rate. The total water loss rate was considered to be 100% in DSP 0, and the rate of weight loss of pots was calculated daily until DSP 6. The soil water loss rate was calculated as the weight loss rate of the empty pots in DSP 0. ImageJ was used to measure leaf area. The PWLR and TR were calculated as follows (Al-Tamimi et al. 2016):

$$\begin{aligned} {\text{PWLR}} & = {\text{total water loss rate}} - {\text{soil water loss rate}}\;{\text{TR}} \\ & = {\text{PWLR/leaf area (cm}}^{{2}} {)} \\ \end{aligned}$$

### Statistical analysis

A two-way ANOVA test (Sidak’s multiple comparison test) (*P* < 0.01) was performed for the measured values of the parameters of this experiment. In addition, the correlations between NIR intensity and image parameters were analyzed using Pearson’s correlation coefficient (*P* < 0.01). The significance of differences in irrigation numbers and amounts between WT and *osphyb* plants were tested using the *T*-test (*P* < 0.01). In this study, Graphpad prism8 (La Jolla, California, USA, www.graphpad.com) program for window was used for all statistical processing.

## Results

### Optimization of plant growth in response to drought stress

**Fig. 1:**
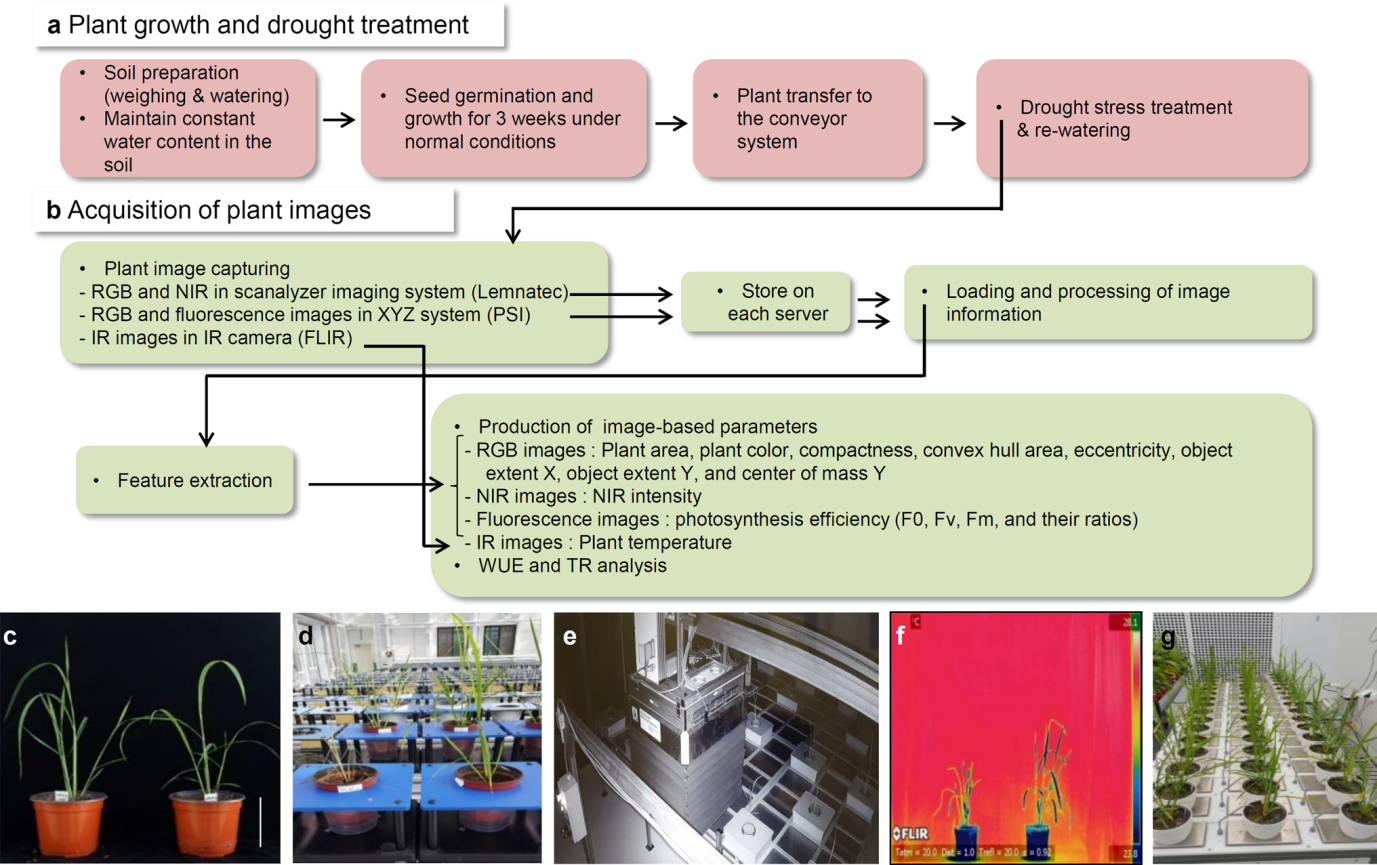
Analysis of plant traits in response to drought stress using plant phenomics technology. **a** Plant growth and drought treatment in the greenhouse. **b** Analysis of drought traits. **c** Plants were grown for 3 weeks in a greenhouse under long-day conditions. **d** Plants were transferred to a conveyor system, and Red–Green–Blue (RGB) and near-infrared (NIR) images were captured under drought stress and after re-watering. **e** Fluorescence imaging. **f** Infrared (IR) imaging. **g** Analysis of water use efficiency (WUE) and transpiration rate (TR) using DroughtSpotter. Left and right images in (**c**), (**d**), and (**f**) show the WT and *osphyb*, respectively. Scale bar = 10 cm

Accurate phenotype analysis requires crops to be cultivated in the absence of additional stresses until the time of imaging. Damage to seedlings caused by increased salinity in the soil can alter the results of the drought stress. To overcome this problem, plants were grown in soil with a salinity of < 0.05%. WT and *osphyb* seedlings were grown in well-watered conditions in a greenhouse for 3 weeks after sowing before drought experiments. After 3 weeks of growth, plants were transferred to a conveyor system and divided into two groups, each containing one-half of the seedlings. One group was grown under normal conditions, and the other group was grown under drought conditions (Fig. 1).

**Fig. 2:**
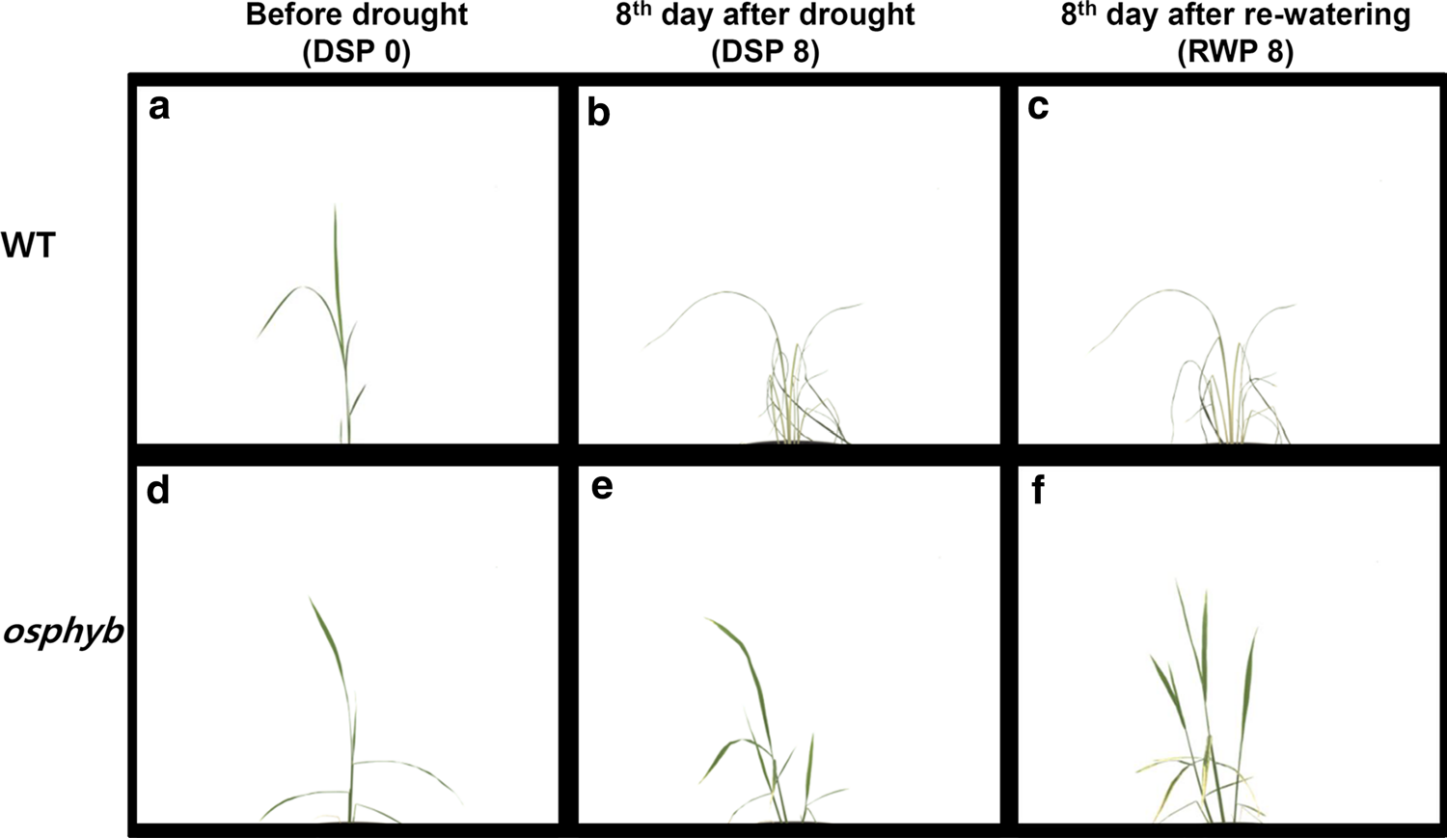
RGB images of WT and *osphyb* plants obtained using image scanners before and after the induction of drought stress and re-watering. **a** and **d** Show images before drought stress induction; **b** and **e** show images obtained in drought stress phase (DSP) 8 (8th day after drought stress); **c** and **f** show images obtained in re-watering phase (RWP) 8 (8th day after re-watering). **a–c** WT images and **d–f** *osphyb* images

The water content of the pots used to grow WT and *osphyb* seedlings was 40–50% at the start of drought stress experiments (Suppl. Fig. S1, and Fig. 2a, d). At 7 days after the induction of drought, the water content of the soil was 0%; WT plants showed leaf wilting, whereas *osphyb* plants did not show obvious morphological changes. After 8 days of drought stress, all plants underwent re-watering for recovery (Fig. 2b, e). After re-watering, the leaves of WT plants did not recover fully, whereas *osphyb* plants continued to grow normally (Figs. 1d, 2c, f).

### Quantitative analysis of drought phenotypes using RGB image-based parameters

**Fig. 3:**
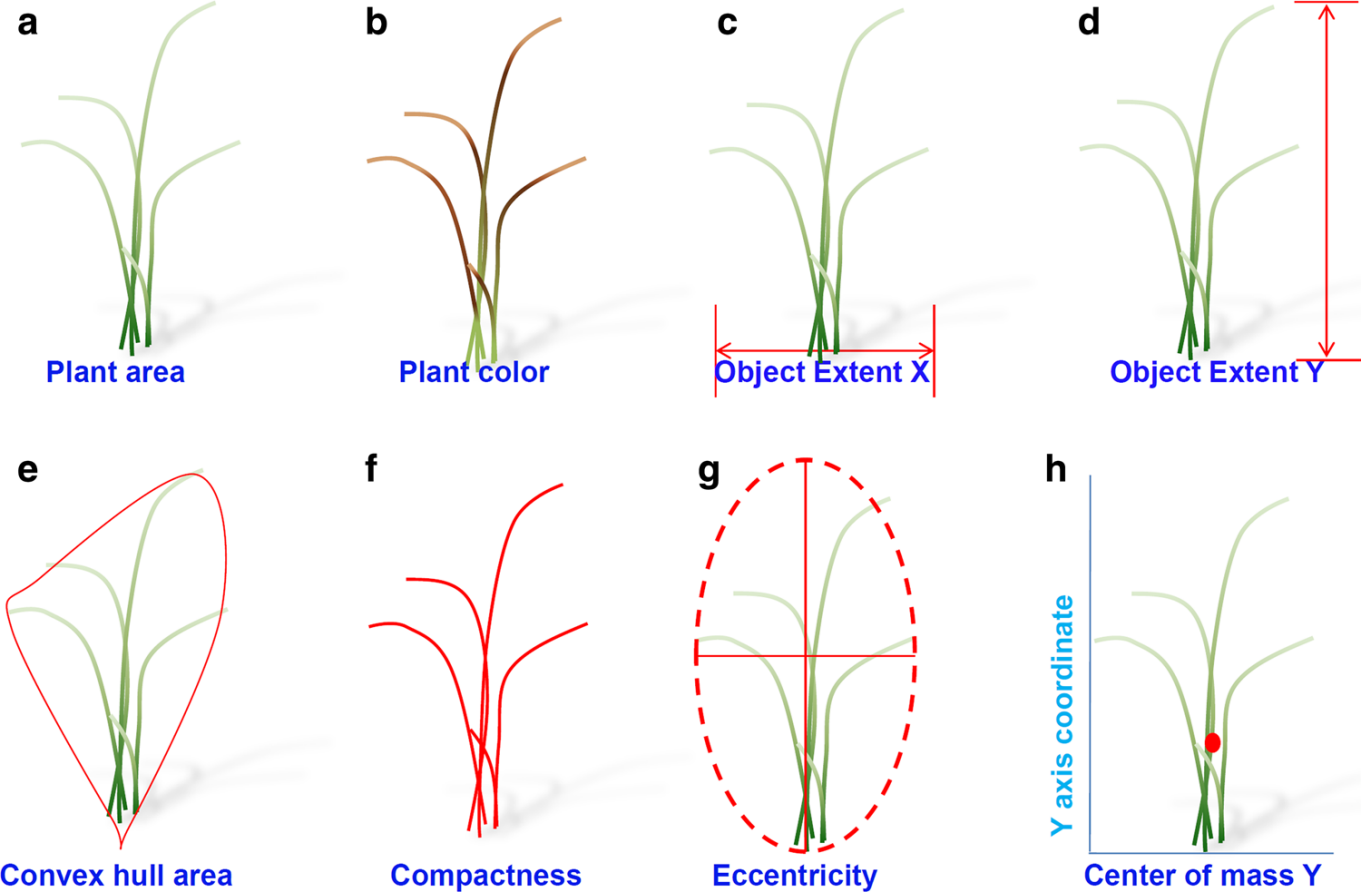
Image-based parameters of drought-related traits determined using RGB imaging. **a** Projected plant area; **b** plant color; **c** object extent X; **d** object extent Y; **e** convex hull area; **f** compactness; **g** eccentricity; and **h** center of mass Y

The parameters that most accurately reflected the morphological characteristics of plants were selected for image analysis in plants exposed to drought stress. The parameters used were biomass, plant color, and morphology (Figs. 1, 2, 3, and Table 1).

**Table 1:** Parameters used to analyze growth and drought-related traits

Types	Description
Plant area	Plant area was represented by the pixel number of the leaf above the plant. Growth rate was obtained by dividing the difference in the number of pixels between the drought stress treatment intervals by stress treatment time (days)
Plant color	Plant color was expressed by extracting the color of the leaf sheath and blade including the stem in Hue. Near-yellow and near-green channels were used for investigating the degree of drought stress
Object extent X	Object extent X indicates the *x*-axis length of the rectangle covering the object and was used to measure plant width
Object extent Y	Object extent Y indicates the *y*-axis length of the least vertical oriented rectangle covering the object and was used to measure the projected plant height
Convex hull area	The convex hull area indicates the smallest area enclosed by the outer contour of an object
Compactness	Compactness is the object area divided by the convex hull area. This can express the density of plants including tillers and leaves
Eccentricity	Eccentricity is a parameter related to the conic section in mathematics. It is defined between 0 and 1, and the shape of the plant is expressed as 0 for a circle and 1 for a line
Center of mass Y	Center of mass Y indicates the center of gravity of the *y*-axis. During the drought stress test, it was used to express leaf drying
Perimeter	The length of the outside boundary of the object
NIR intensity	The water content of the plant was measured by NIR intensity obtained at the water absorption wavelength of 1450 nm
Plant temperature	Plant temperature was extracted from infrared image, which had a resolution of 640 × 480 resolution and a spectral range of 7.5–13 µm
Fluorescence area	Fluorescence area refers to the total area of plants that emitted light during fluorescence measurements. Fv/Fm was calculated from this area
Fv/Fm	Fv/Fm means maximum PSII quantum yield in the dark-adapted state
Water use efficiency	WUE means the value obtained after subtracting the dry weight before and after the drought stress and dividing it by the total irrigated water
Plant water loss rate	This was calculated by subtracting the soil water loss rate from the total water loss rate
Transpiration rate	The transpiration rate is the value obtained by dividing the plant water loss rate by the leaf area

**Fig. 4:**
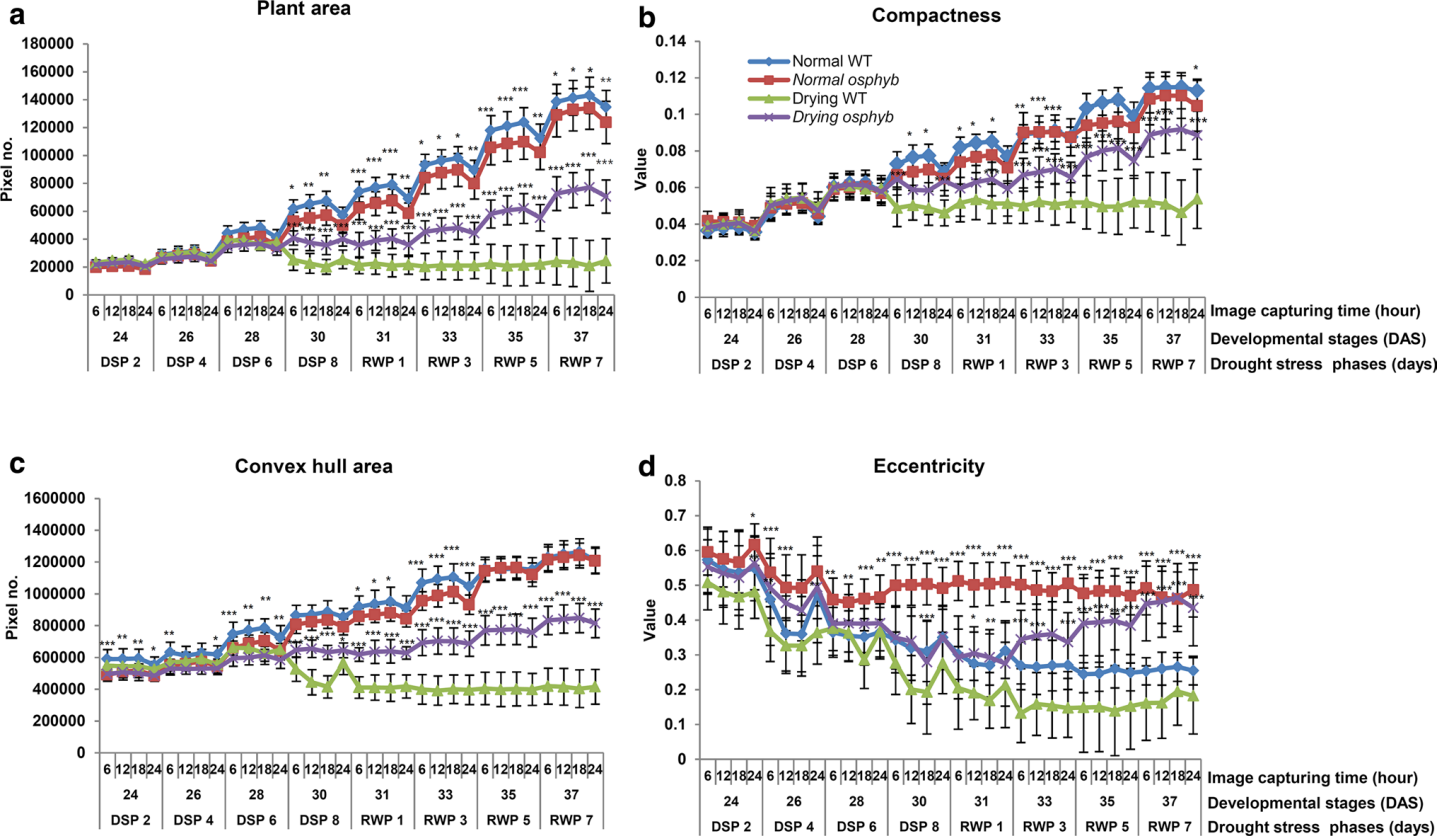
Differences between image-based parameters on the side-view between the WT and *osphyb* plants under normal and drought stress conditions. **a** Projected plant area; **b** compactness; **c** convex hull area; **d** eccentricity. The normal and drought groups were composed of 20 WT and 20 *osphyb* plants each. Statistics analysis of the image parameters was performed using two-way ANOVA test (*P* < 0.01). Each data point is the average of value and measurement, and vertical bars indicate standard deviation. *P < 0.05; **P < 0.01; ***P < 0.001

In plants grown under normal conditions, the projected plant area, compactness, convex hull area, object extent X, and object extent Y increased during the developmental stages in WT and *osphyb* plants (Fig. 4 and Table 1). The projected plant area decreased at 06:00 h, followed by an increase at 12:00 and 18:00 h, and a decrease at 24:00 h at night (Fig. 4a). The overall area increased from about 2.0 × 10^4^–1.4 × 10^5^ pixels. To determine the changes in plant area in the daytime and night-time, we calculated the difference in the projected plant area between 12:00 and 24:00 h. The projected plant areas between WT and *osphyb* plants were increased by approximately 2.0 × 10^3^–9.1 × 10^3^ pixels during the day under normal conditions (Suppl. Table S1). These results suggested that rice leaves expanded during the day to maximize photosynthesis and folded during the night. This patterns was shown similarly in WT and *osphyb* plants, with a variation of about 4.0 × 10^–2^–1.1 × 10^–1^ values for compactness and about 5.0 × 10^4^–1.3 × 10^6^ pixels for convex hull area (Fig. 4b, c, and Table 1). Compactness and convex hull area were used as parameters to estimate the density and spread of the leaves, respectively (Table 1). The average eccentricity of WT and *osphyb* plants were 3.4 ± 0.98 (× 10^–1^)and 5.0 ± 0.39 (× 10^–1^) values, respectively, during the normal growth period; eccentricity was higher in *osphyb* plants than in WT plants, suggesting that *osphyb* plants had an oval shape because leaf wilting was less visible (*P* < 0.01, Fig. 4d and Table 1, Suppl. Table S2). Object extent X was higher in WT than in *osphyb* plants (*P* < 0.01, Table 1 and Suppl. Fig. S2a, Suppl. Table S2). However, the object extent Y and the height of plants did not differ significantly between the two plants (Table 1 and Suppl. Fig. S2b). Under normal conditions, the center of mass Y and the center of gravity were similar between WT and *osphyb* plants (*P* < 0.01, Table 1 and Suppl. Fig. S2c, Suppl. Table S2).

Under the drought stress condition, the projected plant area did not increase during re-watering after drought stress in the WT, whereas it increased in *osphyb* plants during recovery. The projected plant area was average 2.4 ± 1.59 (× 10^4^) pixels for the WT and 7.0 ± 1.19 (× 10^4^) pixels for the *osphyb* plants on RWP 7, showing a 2.9-fold difference. The change of the projected plant area between RWP 1 and RWP 7 was not observed for WT plants. However, *osphyb* plants recovered and showed area changes at the same phases (*P* < 0.01, Fig. 4a, Suppl. Table S2). Only *osphyb* plants showed increases in projected plant area, compactness, and convex hull area (*P* < 0.01, Fig. 4a–c, Suppl. Table S2). The eccentricity was higher in *osphyb* than in WT plants, which could be attributed to plant wilting in the WT during the progression of drought (*P* < 0.01, Fig. 4d, Suppl. Table S2). Regarding object extent X, *osphyb* plants were wider than WT plants, and they showed little changes (*P* < 0.01, Suppl. Fig. S2a, Suppl. Table S2), whereas object extent Y did not differ significantly between WT and *osphyb* plants (*P* < 0.01, Suppl. Figure 2b, Suppl. Table S2). The center of mass Y showed a higher variation of the *y*-axis coordinates in the WT than in the *osphyb* because of leaf wilting (*P* < 0.01, Suppl. Fig. S2c, Suppl. Table S2). Taken together, the results indicate that the RGB image-based parameters that most accurately represented drought variance were projected plant area, compactness, convex hull area, and eccentricity.

### Changes in growth rate induced by drought stress

**Fig. 5:**
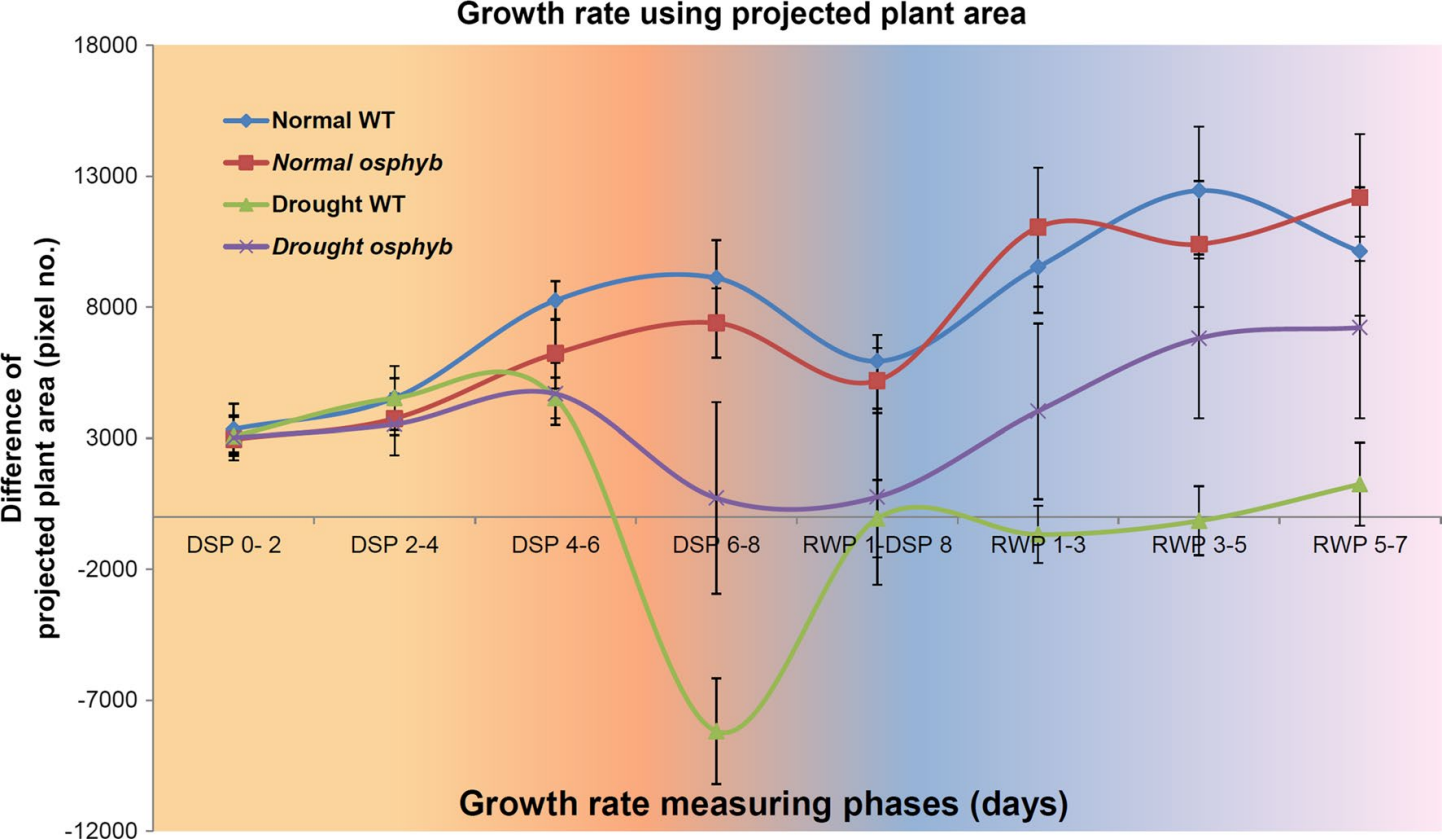
Growth rate analysis using the projected plant area at 12:00 pm under normal and drought stress conditions. The normal and drought groups were composed of 20 WT and 20 *osphyb* plants each. Each data point is the average of growth rate, and vertical bars are standard deviation. *P < 0.05; **P < 0.01; ***P < 0.001

Growth rate was calculated to investigate the effects of drought on plant growth. The results showed that WT and *osphyb* plants grew at similar rates under normal conditions. However, under drought stress conditions, the drought-tolerant *osphyb* plants were decreased to about 4.0 × 10^3^ pixels from DSP 4–6 (i.e., growth rate between 4 and 6 days after drought stress) to DSP 6–8 (i.e., growth rate between 6 and 8 days after drought stress), whereas the drought-susceptible WT plants decreased to about − 1.3 × 10^4^ pixels in the same phases. After re-watering, WT plants did not recover easily, whereas *osphyb* plants showed a gradual recovery (Fig. 5). These results indicate that changes in growth rate are adequate indicators of plant growth in response to drought stress.

### Changes in plant color induced by drought stress

**Fig. 6:**
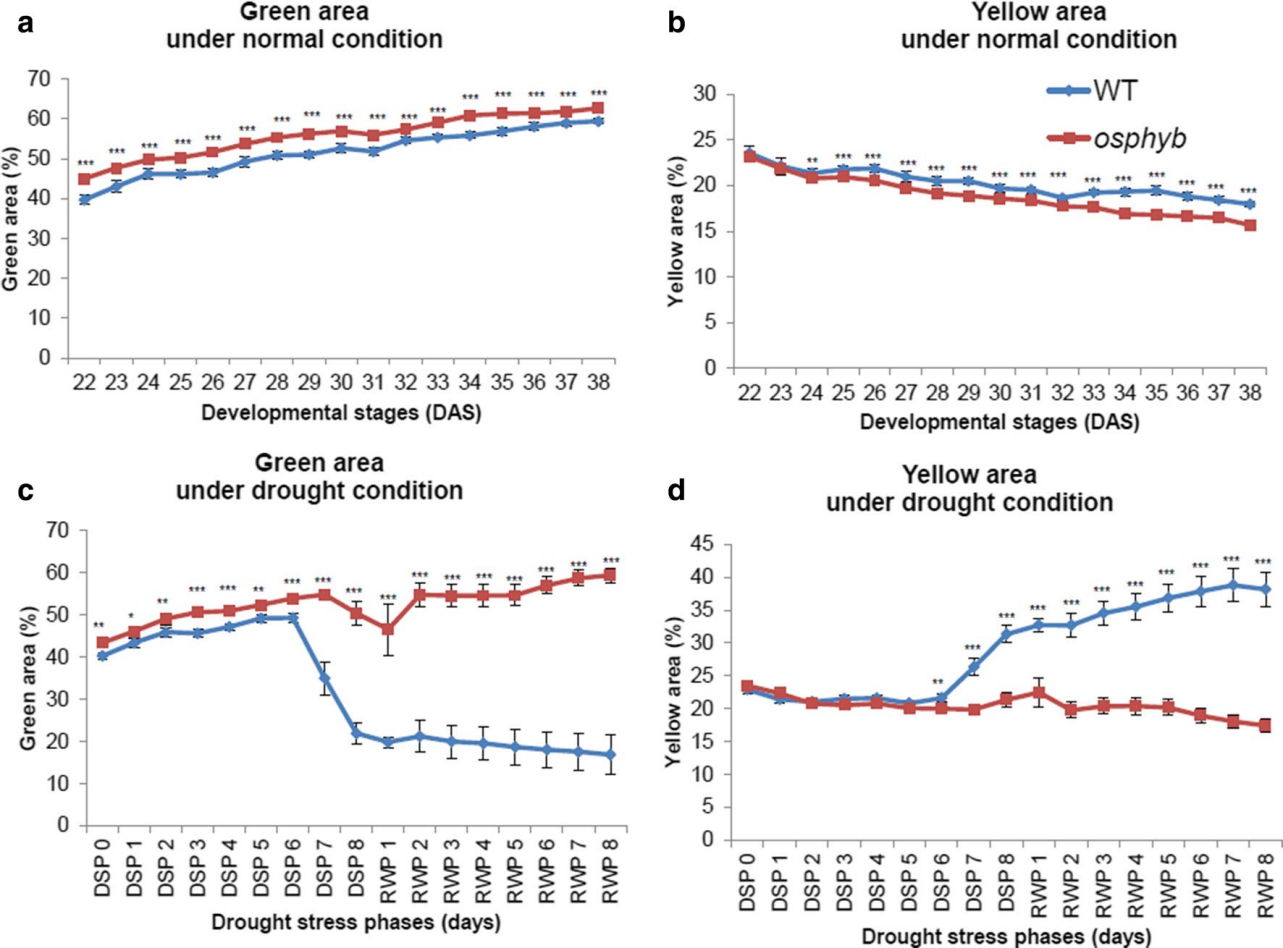
Differences in the plant color areas at 12:00 pm from a side-view. **a** Green area under normal conditions. **b** Yellow area under normal conditions. **c** Green area under drought conditions. **d** Yellow area under drought conditions. The normal and drought groups were composed of 20 WT and 20 *osphyb* plants each. Statistics analysis of plant color areas was performed using a two-way ANOVA test (*P* < 0.01). Each data point is the average of measurement, and vertical bars indicate standard deviation. *P < 0.05; **P < 0.01; ***P < 0.001

Changes in plant color under drought and normal conditions were assessed by measuring the number of pixels in the near-green and near-yellow regions in the Hue channels (Fig. 6 and Suppl. Fig. S3). The near-green region reflects normally growing plant areas, whereas the near-yellow region indicates discoloration of the leaf sheath, leaf blade, and stem caused by drought stress. This color separation was analyzed using histograms dividing the HSI-Hue channel into 0–180 areas under normal and drought stress conditions (Fig. 6 and Suppl. Fig. S3). Under normal conditions, the histogram did not differ significantly between WT and *osphyb* plants (*P* < 0.01, Suppl. Fig. S3a–c, Suppl. Table S2). Under drought stress conditions, the color regions were classified in the near-yellow in the 0–72 region and the near-green in the 73–180 region of the Hue channel (Suppl. Fig. S3 d–f). WT and *osphyb* plants showed different peaks. Under normal conditions, the proportion of near-green regions increased gradually, whereas that of near-yellow regions decreased in WT and *osphyb* plants on 24–37 DAS (day after sowing) (*P* < 0.01, Fig. 6a, b, Suppl. Table S2). The proportion of near-green regions was 3–5% higher in *osphyb* plants than in WT plants (*P* < 0.01, Fig. 6a, Suppl. Table S2), which appeared to be a phenotypic feature of the *osphyb* plants.

As drought stress progressed, the proportion of near-green regions decreased, and that of near-yellow regions gradually increased because of leaf wilting and discoloration of the leaf in the WT compared with the *osphyb* (*P* < 0.01, Fig. 6c, d, Suppl. Table S2). The near-yellow region accounted for approximately 31 ± 1.4% of the total projected plant area in the WT and approximately 21 ± 1.1% in the *osphyb* plants in DSP 8, indicating that WT plants had an approximately 10% larger near-yellow area (*P* < 0.01, Fig. 6d, Suppl. Table S2). The near-yellow region represented the stressed area of plants, whereas the near-green region indicated normal growth. The color classification of RGB images was an effective parameter to analyze the drought characteristics of rice.

### Correlation of NIR intensity with other image-based parameters under drought conditions

**Fig. 7:**
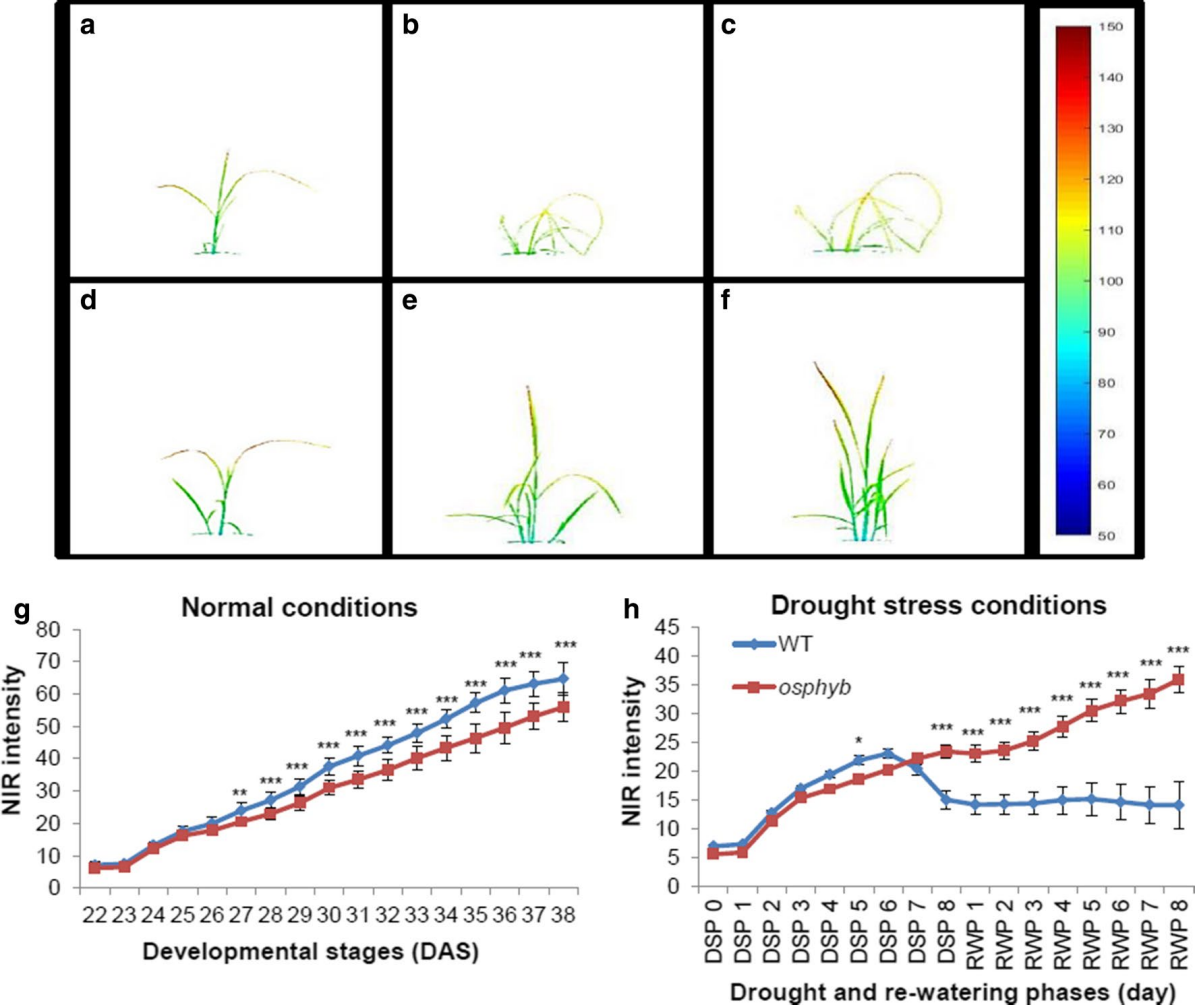
Drought responses of WT and *osphyb* plants at 12:00 pm determined using NIR imaging. **a**, **b**, and **c** represent the WT; **d**, **e**, and **f** represent the *osphyb*. **g**, **h** NIR intensities under normal and drought stress conditions. The normal and drought groups were composed of 20 WT and 20 *osphyb* plants each. Statistics analysis of NIR intensity were performed using a two-way ANOVA test (*P* < 0.01). Each data point is the average of measurement, and vertical bars are standard deviation. *P < 0.05; **P < 0.01; ***P < 0.001

**Fig. 8:**
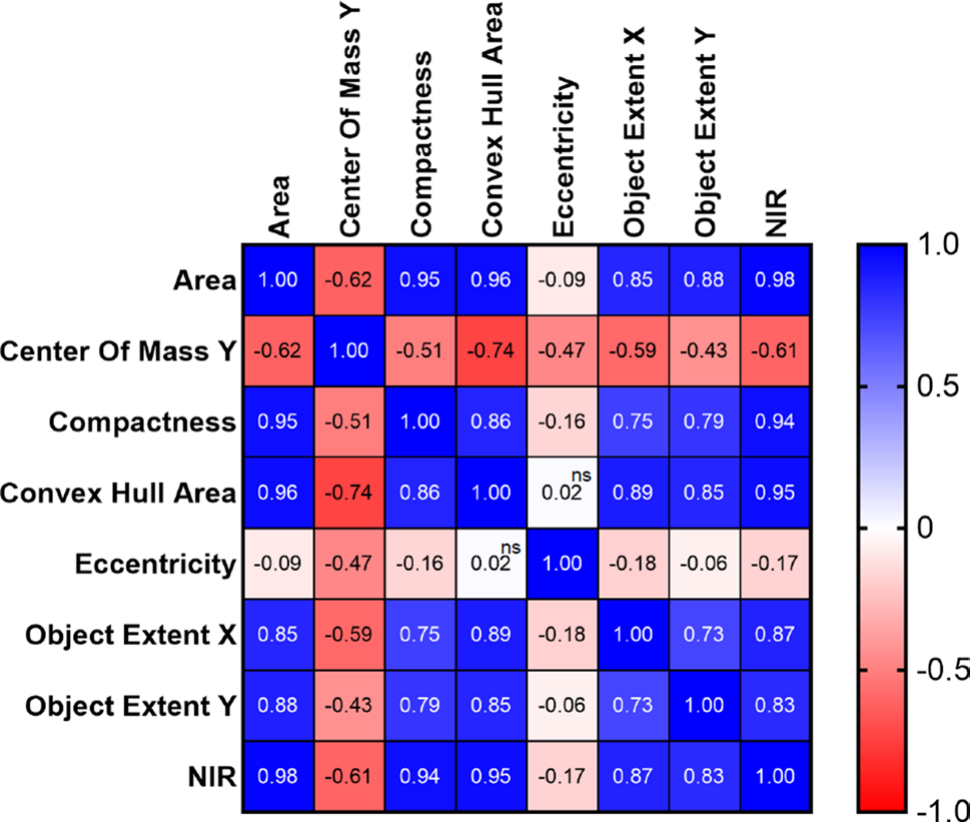
Pearson’s correlation coefficients of image-based parameters and NIR intensity. Statistics analysis of IR measurements was performed using two-way ANOVA test (*P* < 0.01). ns, non-significant

NIR is a commonly used tool for measuring the water content of plants. At 22 DAS, NIR intensity was similar between WT and *osphyb* plants (7.1 ± 0.95 and 6.2 ± 0.72 values, respectively) (*P* < 0.01, Fig. 7a, d, and g, Suppl. Table S2). In DSP 8, NIR intensity differed considerably between WT and *osphyb* plants. In the WT, the amount of water in the plant decreased significantly, whereas *osphyb* plants did not show a large water loss (*P* < 0.01, Fig. 7b, e, and h, Suppl. Table S2). The soil water content of pots was decreased by 0.05% in the WT and by 1.6% in the *osphyb* (*P* < 0.01, Suppl. Fig. S1, Suppl. Table S2). When water was replenished from RWP 1 to RWP 8 and the plants recovered, the NIR intensity of the WT was about 40% that of *osphyb* in RWP 8 (*P* < 0.01, Fig. 7h, Suppl. Table S2). NIR intensity was a good indicator of the stress response, similar to changes in color in response to drought in rice. The projected plant area of the WT was larger than that of the *osphyb* under normal conditions (*P* < 0.01, Fig. 4a, Suppl. Table S2). A similar pattern was observed for NIR intensity (*P* < 0.01, Fig. 7, Suppl. Table S2). In Fig. 8, Pearson’s correlation coefficients between NIR intensity and RGB image parameters were investigated. NIR intensity showed high correlation with plant area, compactness, and convex hull area of 0.98, 0.94, and 0.95 (*P* < 0.01). On the other hand, NIR intensity and center of mass Y and eccentricity showed a low correlation of − 0.61 and − 0.17. As a result, NIR intensity was highly correlated with image-based parameters related to area and density.

### Temperature changes of plants determined using thermal imaging

**Fig. 9:**
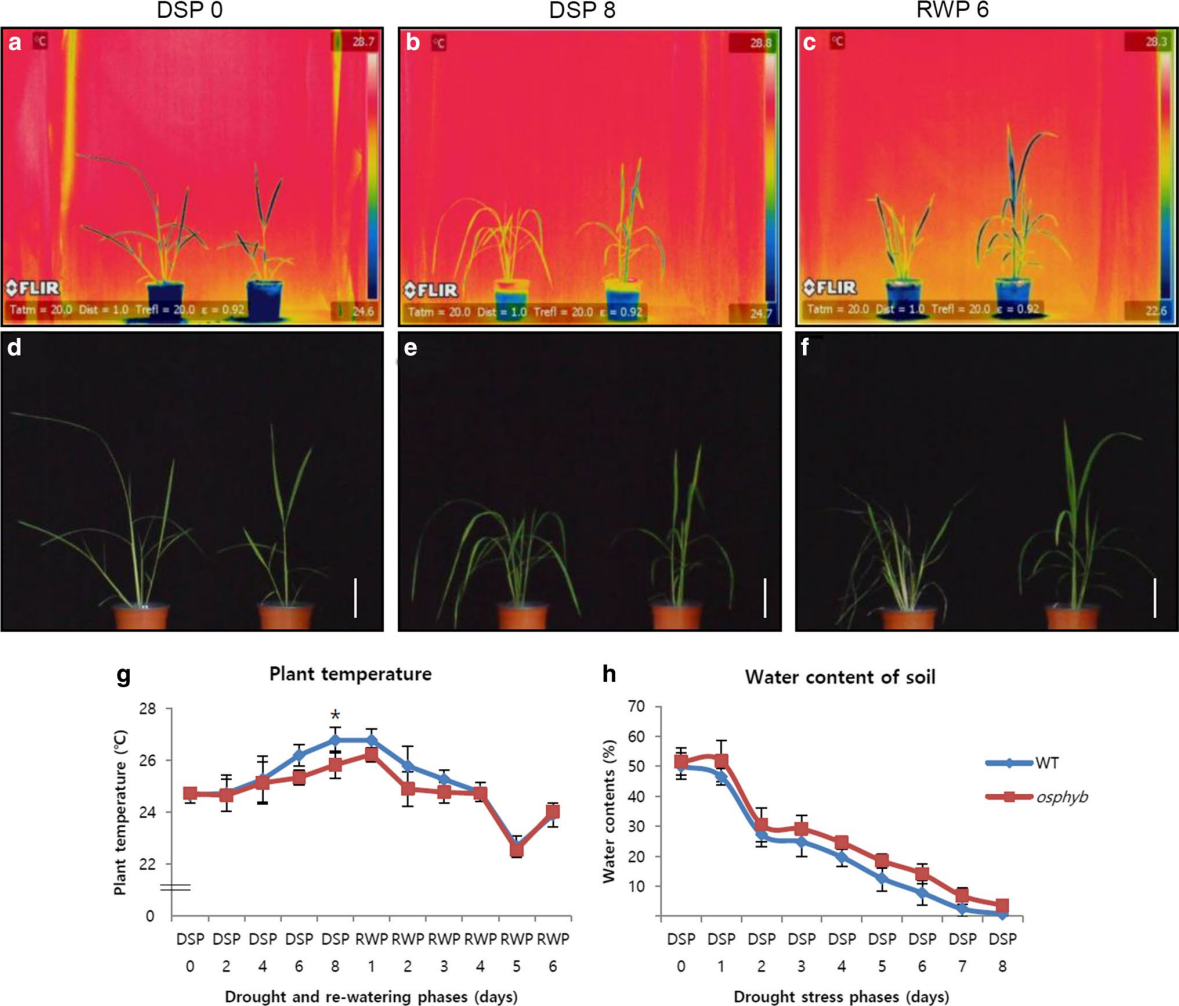
Patterns of plant temperature changes on IR images under drought stress conditions. **a** IR image in DSP 0. **b** IR image in DSP 8. **c** IR image in RWP 6. **d** RGB image in DSP 0. **e** RGB image in DSP 8. **f** RGB image in RWP 6. **g** Plant temperature changes from DSP 0 to RWP 6. **h** Changes in the soil water content during drought and re-watering phases. The plant temperature was determined by calculating the average temperature of each plant. **a**–**f** left plants are the WT and right plants are the *osphyb*. WT, *N* = 5; *osphyb*, *N* = 5. Scale bar = 10 cm. Statistics analysis of IR measurement were performed using two-way ANOVA test (*P* < 0.01). Each data point is the average of measurement, and vertical bars indicate standard deviation. *P < 0.05

The thermal imaging results detected small temperature differences between the leaf blade, leaf sheath, and stem of plants under drought stress conditions. As shown in Fig. 9a, d, and g, the plant temperature was similar between WT and *osphyb* plants in DSP 0 before the induction of drought. In this phase, the average temperature of WT and *osphyb* plants was about 24.7 ± 0.30 °C and 24.7 ± 0.21 °C, respectively, and their temperatures were similar until DSP 4 (Fig. 9g). However, at the DSP 8, the plant temperatures of WT and *osphyb* plants differed by 0.95 °C (*P* < 0.01, Fig. 9g, Suppl. Table S3). At DSP 8, leaf wilting was observed in the WT, and the water content of the soil was 0.74% in the WT and 3.60% in the *osphyb* plants (*P* < 0.01, Fig. 9b, e, and h, Suppl. Table S3). From DSP 0 to DSP 8, the plant temperature changed gradually in response to drought stress in both WT and *osphyb* plants, although WT plants showed a higher temperature (*P* < 0.01, Fig. 9g, Suppl. Table S3). In the re-watering phase, the difference in plant temperature of WT and the *osphyb* showed gradually decreased similarly from RWP 1 to RWP 3, and the temperature was showed no difference in RWP 4–6 (Fig. 9c, f, g). This indicates that it may take up to a minimum of 3 days for plants to recover to some extent after drought stress.

### Analysis of photosynthesis efficiency using fluorescence imaging

**Fig. 10:**
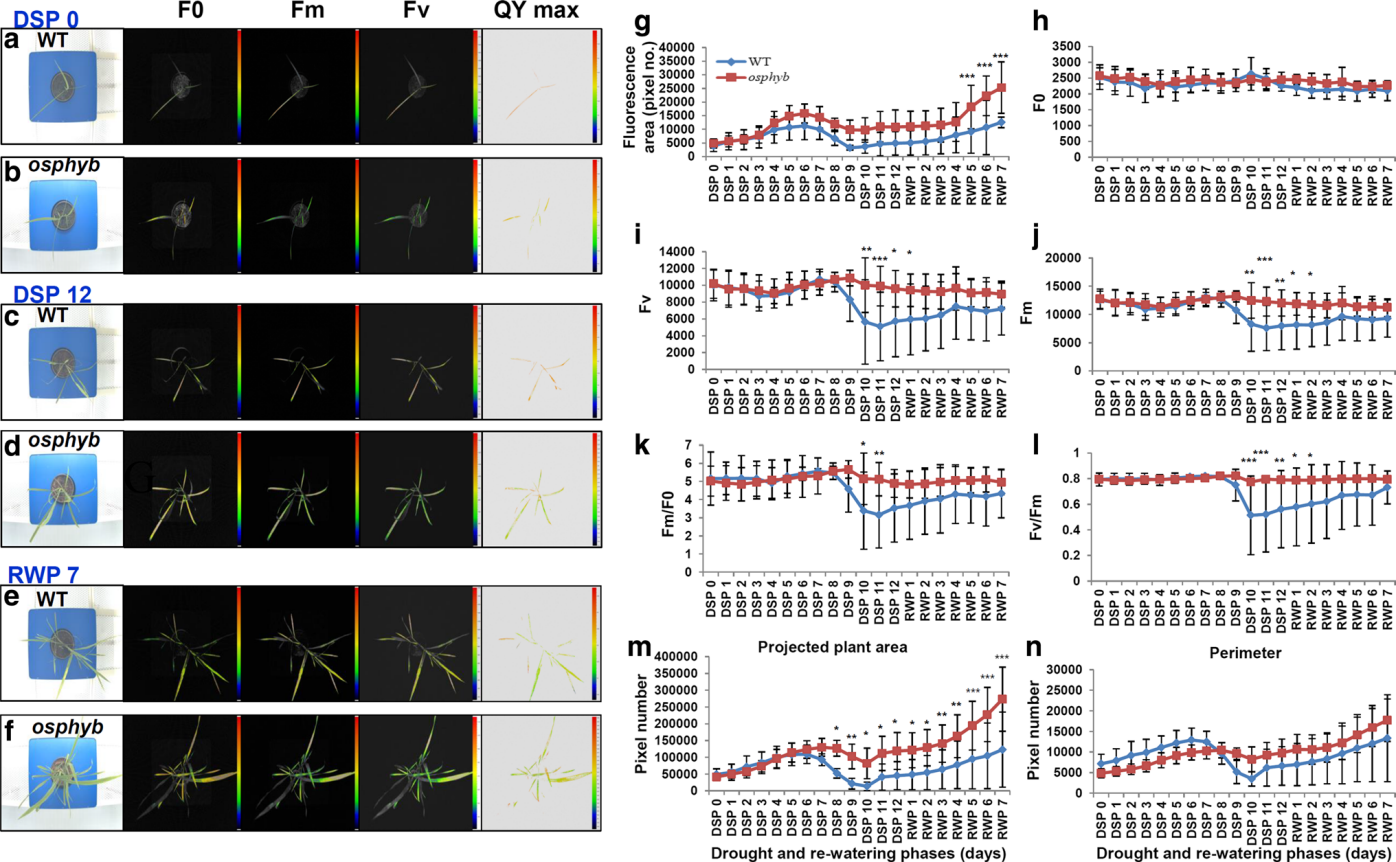
Fluorescence imaging of WT and *osphyb* plants in drought stress and re-watering phases. Images obtained in DSP 0 (**a**, **b**), DSP 12 (**c**, **d**), and RWP 7 (**e**, **f**) are shown. **a**, **c**, and **e** show the WT, and **b**, **d**, and **f** show the *osphyb*. **g** Fluorescence area extracted from fluorescence imaging. **h–i** Indicate F0, Fv, Fm, Fm/F0, and Fv/Fm, respectively. **m** Projected plant area according to RGB parameter on the top-view. **n** Perimeter of RGB parameters on the top-view. WT, *N* = 10; *osphyb*, *N* = 11. Statistics analysis of fluorescence measurements was performed using two-way ANOVA test (*P* < 0.01). Each data point is the average of measurement, and vertical bars are standard deviation. *P < 0.05; **P < 0.01; ***P < 0.001

ChlF analysis is used to analyze the response to drought stress in several plants including rice. However, there are few studies analyzing drought stress and re-watering using fluorescence and RGB parameters from a top-view. To measure the ChlF of rice, the plants were subjected to 12 days of drought stress after 3 weeks of growth under normal conditions, followed by a recovery period of 7 days (Fig. 10). In DSP 0, photochemical parameters including F0, Fv, Fm, and their ratio were similar between the WT and the *osphyb* (*P* < 0.01, Suppl. Table S4). The fluorescence area in DSP 0 was also similar between WT and *osphyb* plants at approximately 3.9 ± 2.02(× 10^3^) and 4.8 ± 1.71(× 10^3^) pixels, respectively (*P* < 0.01, Fig. 10a–l, Suppl. Table S4). In DSP 9, the fluorescence area was approximately threefold larger in WT (~ 3.2 × 10^3^ pixels) than in *osphyb* (~ 9.8 × 10^3^ pixels) plants (*P* < 0.01, Fig. 10g, Suppl. Table S4). Fv, Fm, Fm/F0, and Fv/Fm, but not F0, remained constant in *osphyb* plants, whereas they decreased in WT plants (*P* < 0.01, Fig. 10h–l, Suppl. Table S4). The differences in drought phenotypes between the WT and the *osphyb* were most obvious in DSP 10–12 (*P* < 0.01, Suppl. Table S4). As shown in Fig. 10c, d, leaf wilting and blotting were prominent in WT plants but not in *osphyb* plants. The fluorescence area also differed between the two plants, with mutants showing a larger fluorescence area than WT plants (*P* < 0.01, Fig. 10g, Suppl. Table S4). In RWP 7, *osphyb* plants showed considerable growth, which resulted in a larger fluorescence area than that of the WT. In addition, *osphyb* plants showed higher Fv, Fm, Fm/F0, and Fv/Fm than WT plants. Fm/F0 and Fv/Fm from DSP 0 to RWP 7 remained constant in *osphyb* plants at ~ 5 and 0.8 values, respectively, whereas these ratios decreased to about 3 and 0.5 values in WT plants (Fig. 10k, l). In the top-view RGB, the plant area was comparable to the fluorescence area (Fig. 10m). These two parameters reflected the total area of photosynthesis in the plant canopy. The *R*^2^ between them was 0.93 (*P* < 0.01), which is high (data not shown). The perimeter calculated from RGB data reflected severe patterns of drought. During the early and middle stages of drought (DSP 1–DSP 7), the perimeter was longer in the WT than in the *osphyb* (*P* < 0.01, Suppl. Table S4); however, in DSP 8, the WT did not recover from drought stress, resulting in a decreased perimeter (*P* < 0.01, Suppl. Table S4). This pattern persisted even during the RWP (Fig. 10n). Fluorescence imaging during drought stress was effective for detecting plant stress conditions. The parameters related to ChlF imaging were sensitive, and RGB imaging was a good indicator of the dryness of leaves from the top-view.

### Analysis of WUE, PWLR, and TR under drought stress conditions

**Fig. 11:**
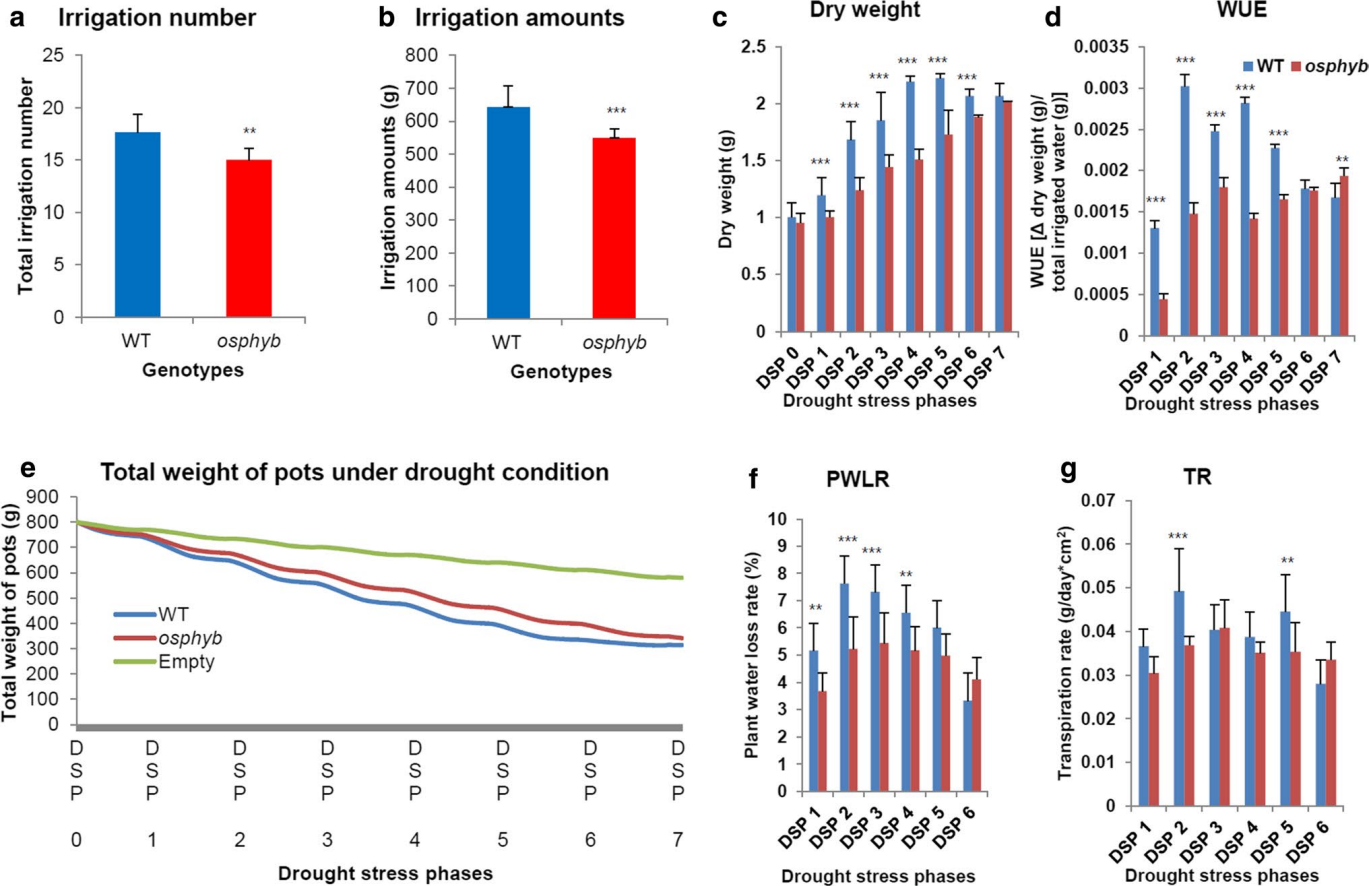
WUE and TR analysis under drought stress conditions. **a** Irrigation number and **b** irrigation amounts at a water shortage of 5%. **c** Dry weight during drought stress. **d** WUE change patterns during drought stress. **e** Total weight changes of empty pots (plant-free pots) and pots with plants (pots of the WT and the *osphyb*). **f** PWLR during drought stress phases. **g** TR during drought stress phases. WT, *N* = 20; *osphyb*, *N* = 20. Statistics analysis of irrigation number and amounts was performed using the *T*-test (*P* < 0.01). Dry weight, WUE, PWLR, and TR were performed using two-way ANOVA test (*P* < 0.01). Each data point is the average of measurement, and vertical bars indicate standard deviation. **P < 0.01; ***P < 0.001

DroughtSpotter was used to determine water consumption in *osphyb* plants (Fig. 11). In the ‘deviation mode’, WT and *osphyb* plants were grown under long-day conditions for 3 weeks in pots filled with water to a weight of 800 g. When the pot weight decreased to 5%, water was replenished. The average irrigation times in the deviation mode were ~ 18 ± 1.8 in the WT and ~ 15 ± 1.1 in the *osphyb* plants (*P* < 0.01, Fig. 11a, Suppl. Table S5). The amount of water used for irrigation in each plant was ~ 643 ± 63.7 g for the WT plants and about 549 ± 28.4 g for the *osphyb* plants, and the difference between them was about 14.6% (*P* < 0.001, Fig. 11b, Suppl. Table S5). Before drought stress (DSP 0/22 DAS), the dry weight was 1.01 ± 0.125 g in WT plants and 0.96 ± 0.085 g in *osphyb* plants. Under drought stress conditions, the dry weight was higher in the WT plants than in the *osphyb* plants (*P* < 0.01, Fig. 11c, Suppl. Table S6). Based on these results, the WUE was measured from DSP 1 to DSP 7. The WUE was calculated by measuring the difference between the dry weight before and after drying, and dividing it by the total irrigated water. The average WUE in DSP 1 was ~ 1.3 ± 0.90(× 10^–3^) in the WT and ~ 4.4 ± 0.70(× 10^–4^) in the *osphyb*, indicating that the WUE was approximately 69.3% higher in the WT than in the *osphyb*. In DSP 2, the average WUE was 3.0 ± 0.14(× 10^–3^) in the WT and 1.5 ± 0.13(× 10^–3^) in the *osphyb*, indicating that the WUE was approximately 50% higher in the WT. However, during the progression of drought stress, the WUE decreased significantly in both WT and *osphyb* plants (*P* < 0.01, Fig. 11d, Suppl. Table S6). Low WUE values indicated increased resistance to drought stress, as less water was used for plant growth.

In the ‘none mode’, the pots of 3-week-old plants weighed 800 g. After the supply of water was terminated, the pots were weighed at 1 min intervals. The empty pots, which were plant-free and contained only soil, were weighed at the same time as pots of WT and *osphyb* plants. In the DSP 7, the average weight of the pots was about 314 g for the WT and about 343 g for the *osphyb* plants, and the difference was about 29 g. The difference in the irrigation water between the two groups of pots was ~ 8.5%. At DSP 7, the average weight of empty pots was ~ 581 g (Fig. 11e). In ‘none mode’, WT plants consumed more water than *osphyb* plants. WT plants showed a higher PWLR than *osphyb* plants in DSP 1–5. The biggest difference was observed in DSP 2, with a PWLR of 7.6 ± 0.60% for the WT and 5.2 ± 1.16% for the *osphyb*. At DSP 6, WT leaves showed a higher degree of drying, which resulted in a lower PWLR than that of the *osphyb* (*P* < 0.01, Fig. 11f, Suppl. Table S6). Also, the TR from DSP1 to DSP5 was higher in the WT than in the *osphyb*. At DSP 6, the drought effect was highest, and the TR was slightly higher in *osphyb* plants than in WT plants (*P* < 0.01, Fig. 11g, Suppl. Table S6). During this phase, WT plants were severely drought-stressed and could not normally transpiration, so TR may be lower than that of *osphyb* plants. Eventually, these results showed that loss of function of *OsPhyB* results in lower WUE, PWLR and TR, indicating that *osphyb* plants are resistant to drought.

## Discussion

### Application of RGB image-based parameters of drought stress

Many studies investigated the responses of plants to various stresses such as drought, salt, and high and low temperatures using image analysis (Jones 2002; Roy et al. 2011; Yonemaru and Morita 2012; Hairmansis et al. 2014; Campbell et al. 2015; Humplík et al. 2015). In this study, several RGB parameters including projected plant area, compactness, convex hull area, center of mass Y, and plant color were used to analyze the response of plants to drought stress (Figs. 3, 4, 5, 6, Suppl. Fig S2, Table 1). The results showed that the most effective indicators of drought-tolerant phenotypes were projected plant area, plant color, compactness, convex hull area, and eccentricity (Gupta et al. 2012; Deshmukh et al. 2014; Kumar et al. 2015; Malinowska et al. 2017).

The projected plant area was previously used to represent the biomass of plants in many crops, such as corn, barley, and wheat (Yang et al. 2009, 2013; Golzarian et al. 2011; Mir et al. 2019). In this study, the side-view in Fig. 4a shows that under normal conditions, the projected plant area increased in WT and *osphyb* plants rhythmically in the day and night. Similar results were reported in *Phaseolus coccineus* (McClung 2006). These results, which were not previously reported in monocotyledonous plants such as rice, represent morphological changes that increase the projected plant area during the daytime to improve the photosynthetic efficiency of rice. When viewed through image parameter analysis, morphological changes due to drought stress began to show a difference between WT and *osphyb* plants starting from DSP 6. It was closely related to changes in soil water content. For the most plants, soil water content was decreased below 10% in DSP 4. At DSP 6, soil water content for WT plants was ~ 0.7% and that for *osphyb* plants was ~ 2.4%, which indicates that there was little moisture in the soil of WT plants. After DSP 6, leaf wilting started to occur in WT plants.

### Correlation between water content and image area in plants determined by NIR imaging

The main response of plants to drought stress was a reduction in total plant area resulting in a decrease in the water content of plants. The correlation between the projected plant area and NIR intensity was an indicator of the water content of plants. Pearson’s correlation coefficient (*R*) was showed between the plant area and NIR intensity was 0.98 (*P* < 0.01) (Fig. 8). NIR intensity was also highly related to another plant area-related parameter, namely, convex hull area. NIR intensity was identified as an optimal measure for determining biomass changes in plants under drought stress conditions. NIR imaging could be a useful tool to detect drought-related traits in rice. Li et al. (2001) analyzed the plant fresh biomass and NIR reflectance of cotton and showed that the two parameters are positively correlated, with a high *R*^2^ of 0.93 (*P* < 0.01). NIR imaging was also effective for measuring the water content of rice seeds. At a wavelength of 1450 nm, the *R*^2^ between NIR reflectance and the water content of rice seeds was 0.936 (Lin et al. 2019).

### Plant temperature changes determined by IR imaging

IR thermography is used to analyze the responses of crops to abiotic or biotic stresses (Stoll and Jones 2007; James and Sirault 2012; Ballester et al. 2013). Kwon et al. investigated the role of IR imaging in the response to stress in a drought-tolerant transgenic line of rice expressing *CaMsrB2* (Siddiqui et al. 2014; Kwon et al. 2015). In this study, we examined the changes in plant temperature in response to drought stress (Fig. 9). Plant temperature was lower in the drought-tolerant *osphyb* plants than in the WT plants. This could be associated with the higher water content in *osphyb* plants than in WT plants. In addition, NIR intensity was higher than that of *osphyb* under normal conditions, as shown in Fig. 7g, h. The water content of the WT decreased significantly after 8 days of drying. Another possible explanation for the temperature differences suggested by IR images it the stomatal pore structure and TR. Stomatal density, stomatal size, net CO_2_ uptake, and TR are lower in *osphyb* than in WT plants, as reported previously (Liu et al. 2012). A lower stomatal density and size may lead to a decrease in transpiration and a lower rate of water loss under drought stress. Leaf stomatal density is correlated with stomatal conductance, net CO_2_ assimilation, and WUE. In addition, the leaf water potential increases with increasing stomatal size (Xu and Zhou 2008). A comparison of *STOMAGEN* overexpressing (ST-OX) and silenced (ST-RNAi) lines with WT (CS60000) in *Arabidopsis thaliana* showed that the TR was 14% higher in ST-OX, which has high stomatal density, and 34% lower in ST-RNAi, which has low stomatal density, than that in the WT (Larcher et al. 2015).

### Analysis of photosynthesis efficiency using fluorescence imaging

ChlF is a key indicator of the growth and photosynthesis efficiency of crops (Baker and Rosenqvist 2004; Narayan et al. 2012; Murchie and Lawson 2013). Fluorescence parameters, including Fv, Fm, Fm/F0, and Fv/Fm, are used to determine the status of crops in response to various stresses (Baker and Rosenqvist 2004; Adams and Demmig-Adams 2007; Rousseau et al. 2013). In this study, the Fv/Fm ratio, which indicates photochemical efficiency, did not change significantly in early response for drought stress (Fig. 10l). Rice has several phytochrome subfamilies, such as *OsPhyA*, *OsPhyB*, and *OsPhyC*, which may be related to functional redundancy (Takano et al. 2005, 2009; Jumtee et al. 2009). Loss of *OsPhyB* may be compensated by other genes, and the efficiency of photosynthesis thus remains constant. Fv/Fm remained constant until DSP 8 in the *osphyb* plants, whereas it decreased considerably in the WT (Fig. 10l). This could be attributed to the fact that photoinhibition caused by water deficiency and inactivation of photosynthesis-regulating enzymes occurred at a faster rate in WT than in *osphyb* plants (Zlatev 2009; Gururani et al. 2015; Wang et al. 2018).

### Analysis of WUE, PWLR, and TR under drought stress conditions

We described the *osphyb* phenotypes determined by RGB, IR, NIR, and fluorescence imaging. Water use, loss, and transpiration are important factors. As shown in Fig. 11, the *osphyb* plants required less water than the WT, and had lower WUE, PWLR, and TR values, which may reflect differences in plant biomass between the two genotypes. The dry and fresh weight measurements of plants also indicated differences in biomass between WT and *osphyb* plants (Fig. 11c and Suppl. Figure 4). A greater biomass requires more water for survival and photosynthesis.

In Fig. 11c, the difference in WUE is greater than the difference in dry weight between the WT and the *osphyb*. This suggests that the tolerance traits of the *osphyb* plants are complex and involve other factors in addition to biomass. The *osphyb* plants has a lower stomatal density and length than the WT (Liu et al. 2012), and the lower number and size of stomata per unit area may affect the TR, which may lead to differences in WUE. Another difference between the two plants is root length and biomass. Root length and dry weight are decreased in the *osphyb* plants under normal conditions (Liu et al. 2012; Yoo et al. 2017). The morphological and physiological effects of the mutation on the roots may have contributed to the reduction of WUE because they reduced the ability of the *osphyb* to absorb water from the soil. As shown by IR imaging, the low stomatal density of the *osphyb* may lead to a decrease in WUE, PWLR, and TR (Fig. 11). In this study, we investigated rice drought-tolerant phenotypes using image-based phenotyping and various image measuring instruments. The results showed that imaging methods can be used to detect small phenotypic changes and assess WUE and TR to test the drought tolerance of rice.

RGB image analysis is a time- and cost-effective technique. Image-based parameters related to area and color changes showed the highest efficacy for the quantification of morphological traits. NIR, IR, and fluorescence imaging were identified as effective methods for the analysis of plant phenotypes because of their ability to quickly visualize and quantify the water content, temperature, and photosynthetic efficiency of plants. The physiological responses of plants to drought were quantified by measuring WUE and TR using DroughtSpotter, which helped to characterize the actual drought resistance of the mutants. In the future, our platform could be used to screen large numbers of drought-tolerant rice cultivars through automation and advances in image analysis technologies.

#### *Author contributions Statement*

SK wrote the manuscript and designed and conducted all the experiments. NK arranged and operated RGB imaging, fluorescence imaging, and the DroughtSpotter system. HL performed image processing for RGB, NIR, and IR. EL and KC performed greenhouse management for the experiment, and participated in discussions regarding the manuscript. MK performed statistical analysis. JB operated RGB imaging software and fluorescence imaging systems. IC managed and operated the high-throughput phenotyping systems. HJ and IY helped to write and discuss the manuscript. KJ provided materials and participated in discussions regarding experiments. TK designed the project and discuss the manuscript. KK designed the project, supervised all experiments, and reviewed the manuscript. All authors approved the content of the final manuscript.

## Electronic supplementary material

Below is the link to the electronic supplementary material.

Supplementary file1 (PDF 134 kb)

Supplementary file2 (XLSX 38 kb)

## Abbreviations

RGB: Red–Green–Blue
NIR: Near-infrared
IR: Infrared
WUE: Water use efficiency
TR: Transpiration rate
ChIF: Chlorophyll fluorescence
DSP: Drought stress phase
RWP: Re-watering phase
PWLR: Plant water loss rate
