# Supplementary material for: High-throughput phenotyping platform for analyzing drought tolerance in rice
Source: Planta. 2020 Aug 10;252(3):38. doi: 10.1007/s00425-020-03436-9 (PMC7417419; doi:10.1007/s00425-020-03436-9)
Supplement: Supplementary file 1 — Supplementary file1 (PDF 134 kb) [file 425_2020_3436_MOESM1_ESM.pdf]

Journal name : Planta

High-throughput phenotyping platform for analyzing drought tolerance in rice

Song Lim Kim[1], Nyunhee Kim[1], Hongseok Lee[1, 2], Eungyeong Lee[1,3], Kyeong-Seong Cheon[1], Minsu Kim[1], JeongHo Baek[1], Inchan Choi[1], Hyeonso Ji[1], In Sun Yoon[1], Ki-Hong Jung[4], Taek-Ryoun Kwon[1] and Kyung-Hwan Kim[1*]

[1]The National Institute of Agricultural Sciences, 370 Nongsaengmyeong-ro, Wansan-gu, Jeonju-si, Jeollabuk-do, Republic of Korea
[2]Department of Agricultural Machinery Engineering, Chungnam National University, Daejeon 34134, Republic of Korea
[3]Department of Crop Science and Biotechnology, Jeonbuk National University, Jeonju 54896, Republic of Korea
[4]Graduate School of Biotechnology & Crop Biotech Institute, Kyung Hee University, Yongin, Republic of Korea

Running head: Rice platform for drought phenotyping

*Corresponding author: Kyung-Hwan Kim, biopiakim@Korea.kr
Tel: +82-63-238-4658
Fax: +82-63-238-4654

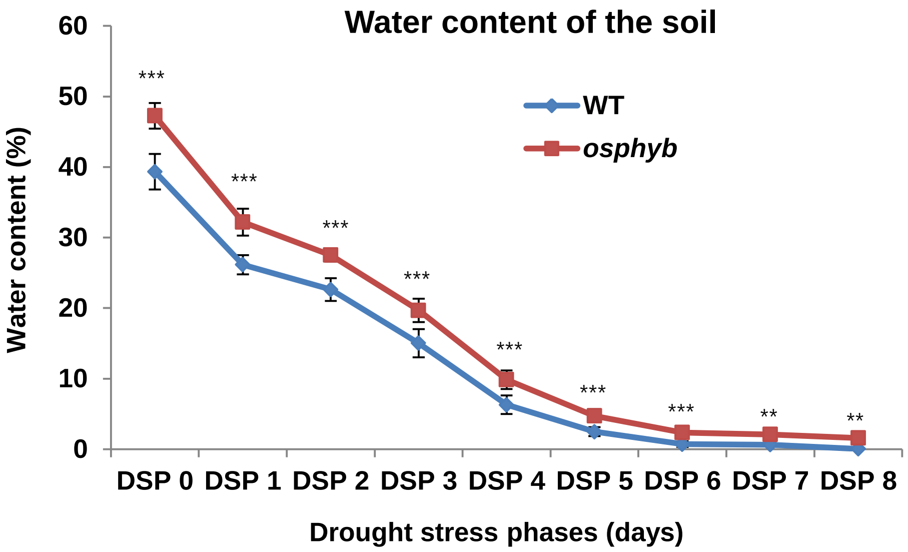

Suppl. Fig. S1. Water content (%) of the soil on RGB and NIR imaging during drought stress phases. DSP means drought stress phases. Each data point is the average of value and measurement, and vertical bars are standard deviation. **, $P<0.01$; ***, $P<0.001$

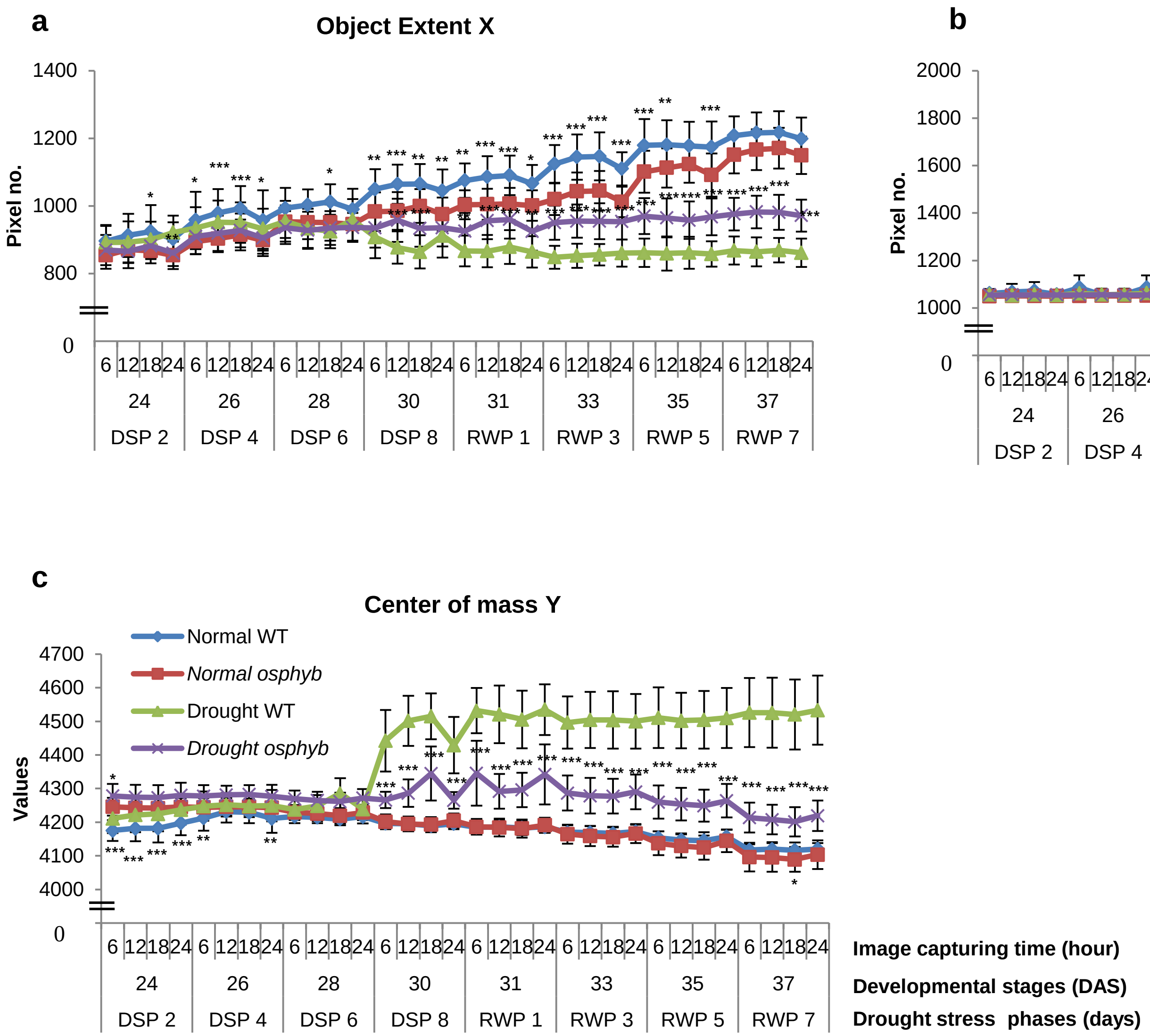

Suppl. Fig. S2. Changes of object extent X (a), object extent Y (b), and center of mass Y (c) on the side-view under normal and drought stress conditions. The statistics analysis of image parameters were performed by a two-way anova test (P<0.01). Each data point is the average of measurement, and vertical bars are standard deviation. *, P<0.05; **, P<0.01; ***, P<0.001.

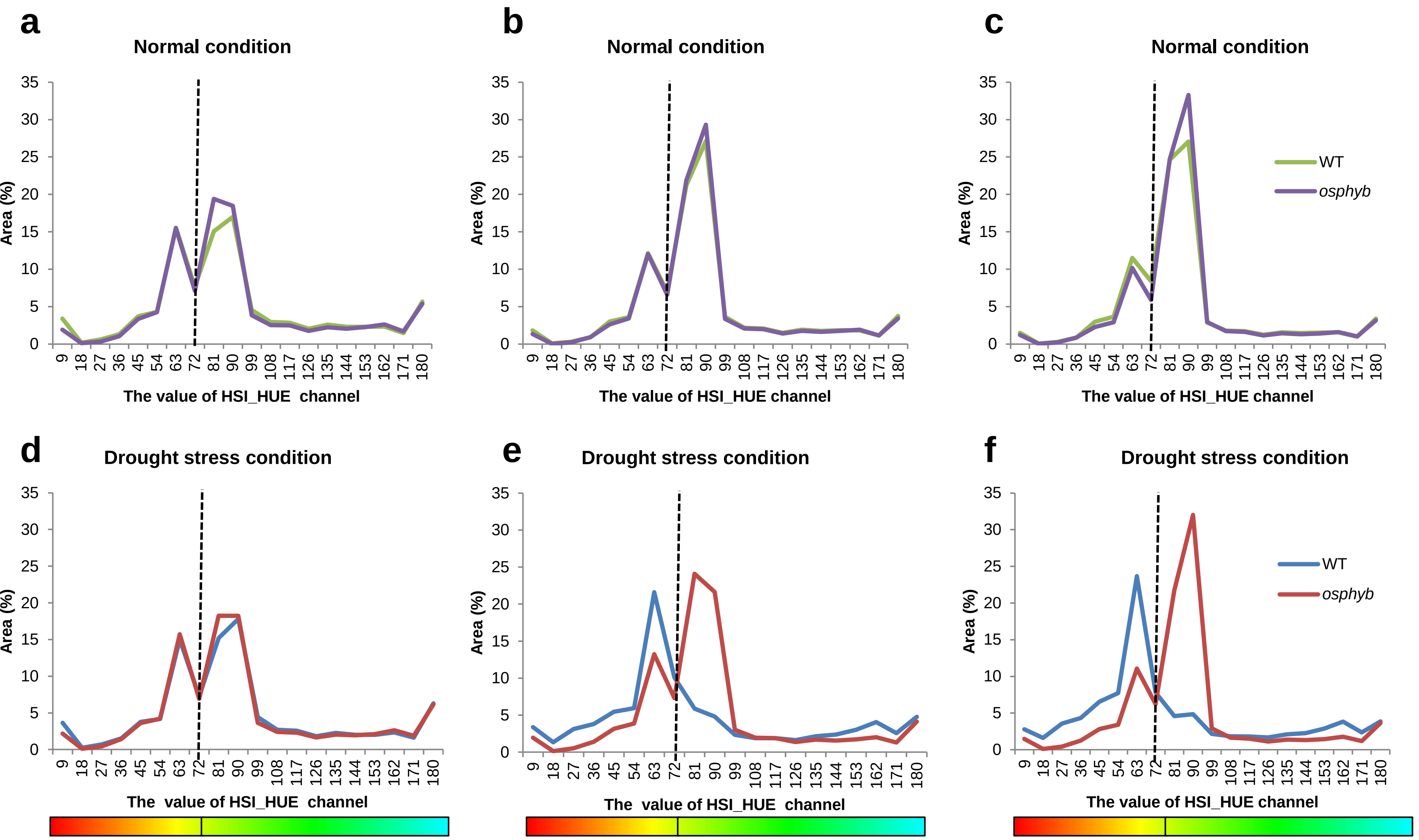

Suppl. Fig. S3. The HUE histograms of normal and drought stressed plants.
The Hue histograms of 22 DAS (a), 30 DAS (b), and 38 DAS (c) plants under normal conditions. The Hue histograms of DSP 0 (d), DSP 8 (e), and RWP 8 (f) plants under drought stress conditions. The HUE histograms were extracted from capturing images at the 12:00 h.

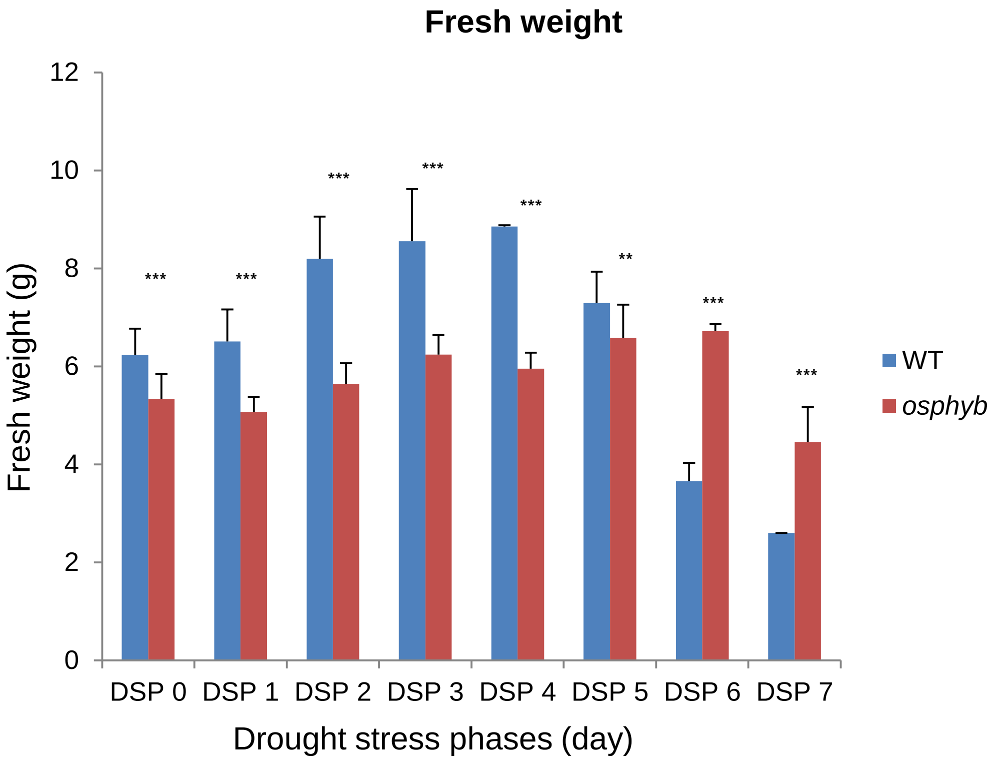

Suppl. Fig. S4. Fresh weight during drought stress phases. DSP means drought stress phases. The statistics analysis of fresh weight were performed by a two-way anova test ($P<0.01$). Asterisks indicate significant differences. **$P<0.01$, ***$P<0.001$. Each data point is the average of value and measurement, and vertical bars are standard deviation. **, $P<0.01$; ***, $P<0.001$.
